# Flow visualization over a thick blunt trailing-edge airfoil with base cavity at low Reynolds numbers using PIV technique

**DOI:** 10.1007/s12650-016-0405-3

**Published:** 2016-11-28

**Authors:** Gholamhossein Taherian, Mahdi Nili-Ahmadabadi, Mohammad Hassan Karimi, Mohammad Reza Tavakoli

**Affiliations:** Department of Mechanical Engineering, Isfahan University of Technology, Isfahan, 84156-83111 Iran

**Keywords:** Thick blunt trialing-edge airfoil, Velocity field, Base cavity, PIV, Wind tunnel

## Abstract

**Abstract:**

In this study, the effect of cutting the end of a thick airfoil and adding a cavity on its flow pattern is studied experimentally using PIV technique. First, by cutting 30% chord length of the Riso airfoil, a thick blunt trialing-edge airfoil is generated. The velocity field around the original airfoil and the new airfoil is measured by PIV technique and compared with each other. Then, adding two parallel plates to the end of the new airfoil forms the desired cavity. Continuous measurement of unsteady flow velocity over the Riso airfoil with thick blunt trailing edge and base cavity is the most important innovation of this research. The results show that cutting off the end of the airfoil decreases the wake region behind the airfoil, when separation occurs. Moreover, adding a cavity to the end of the thickened airfoil causes an increase in momentum and a further decrease in the wake behind the trailing edge that leads to a drag reduction in comparison with the thickened airfoil without cavity. Furthermore, using cavity decreases the Strouhal number and vortex shedding frequency.

**Graphical abstract:**

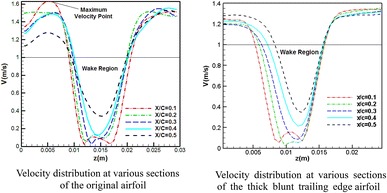

## Introduction

Airfoils with thickened edges are used in subsonic flow to resolve many of the problems encountered in the conventional airfoils. In the conventional airfoils, the trailing edge ends to a sharp point. However, for certain applications (e.g., a wing in high attack angles), an airfoil with a thick trailing edge is required. The gradual reduction of sharpness at the downstream of the maximum thickness section of an airfoil creates a strong positive pressure gradient at its low-pressure side, leading to an untimely flow separation. This positive pressure gradient can be somewhat reduced using a thickened edge. Thus, the recovered pressure can be partially transferred to the separation region produced by the airfoil. Improving the aerodynamic performance of airfoils with thickened edges would require certain drag-reducing instruments (Javarashkian and Lotfi 2008).

Mathey (2008), Heskestad and Olberts (1960), and Zhang et al. (2015) studied numerically, and Bourgoyne et al. (2000), Nakano et al. (2007), and Gerontakos and Lee (2008) studied experimentally the flow characteristics of an airfoil to understand the vortex shedding mechanism.

Cooperman et al. (2010) numerically and experimentally studied the characteristics of UCD-38-095 airfoil with blunt trailing edge. In the experimental part, lift and drag force measurements were carried out in Davis aeronautical wind tunnel at Reynolds numbers of 333,000 and 666,000. Both free and fixed transition conditions were studied. The wind tunnel results are compared with computational predictions obtained in OVERFLOW, a Reynolds-averaged Navier–Stokes solver using structured overset grids.

Baker and Dam (2008) numerically and experimentally studied the FB-3500-1750 airfoil with blunt trailing-edge and used certain instruments to reduce the drag and increase the lift for improving the performance of this airfoil. They used a pyramidal balance for the measurement of the lift and drag at Davis aeronautical wind tunnel. To reduce drag, they implemented two splitter plates as well as open and moderate cavities. Their results showed that using the splitter plates could reduce drag by up to 50%. Although a base cavity reduced drag by up to 25%, it also introduced drastic fluctuations in lift. Using a moderate cavity not only improved the drag-reducing performance of the splitter plate, but also limited the unsteady vortex shedding.

Cai et al. (2008) conducted a numerical study on the sinusoidal edge effect in a flow stopping airfoil-like body with a thick edge to determine how it affected the aerodynamic characteristics of this body. They examined the effect of the sinusoidal edge wavelength on drag coefficient, vortex shedding, and frequency. Their results showed that using a specific wavelength would reduce drag more than 30% as compared with the drag produced by a smooth-edged airfoil. In this case, the smaller separation region resulting from educed drag would lead to a further reduction in the frequency of oscillations inside the separation region as well as increased flow vorticity.

By measuring pressure on the upper surface of an airfoil and implementing the particle image velocimetry (PIV) technique, Aravind and Al-Garni (2009) studied the flow behavior over a two-dimensional bluff body combined to a base cavity with various shapes. For this purpose, they used four types of cavities. Their experiments with the moderate cavity showed that two rotational regions adjacent to the base cavity were created. Moreover, dramatic variations were observed in the length of the recirculation region as well as the mean flow field in the presence of the base cavity.

Ramjee et al. (1986) conducted theoretical and experimental studies on the NACA0012 airfoil with a thick edge. They produced the thick-edged airfoil by cutting off the trailing edge of the airfoil at the following distances: 5, 10, and 15% of the chord length as measured from the end of the airfoil. They calculated the induced lift and drag forces in a wind tunnel at angles between 0° and 20° and *Re* = 400,000. Experiments were conducted in a closed-circuit open-jet wind tunnel with 1.5 m diameter test section at different air speed. Each of the models was attached to a semiautomatic mechanical six component wire balance. The results showed that lift and drag both increased as a result of increasing the trailing edge thickness.

Deshpande and Sharma (2012) empirically studied the effects of connecting trapezoidal prismatic blocks with different sizes to the thick end of an airfoil for the purpose of eliminating Carmen vortices. They used the hot wire apparatus as well as flow detection techniques to conduct their experiments. Their results revealed that using trapezoidal prismatic blocks somewhat weakened the Carman vortices formed at the back of the airfoil. However, pressure distribution measurements showed that drag was reduced by a mere 3–4%.

Olsman (2010) studied the aerodynamic effects of two types of cavity on the NACA0018 airfoil. By flow visualization experiments, they observed vortexes instabilities which produce oscillating forces.

Donelli et al. (2011) numerically investigated the effects of applying suction along the internal points of cavity on the stability of the trapped vortex. They used Fluent software for the numerical simulation.

Gregorio and Fraioli (2008) studied the effect of using circular cavity on a thick blunt trailing-edge airfoil with flow suction, injection, or combination of the two. The pressure distribution along the airfoil and its cavity was obtained using PIV technique. The results showed that using cavity without any suction and injection causes the vortex not to be trapped and again, vortex shedding phenomenon to be generated. However, using suction or injection could control the flow separation in a specified range.

Thao do et al. (2010) studied flow past a blunt-edged two-dimensional NACA 0015 section and the same section with various base cavity shapes and sizes at high Reynolds numbers using the unsteady Reynolds-averaged Navier–Stokes (URANS) approach with the realizable *k*–*e* turbulence model. It is observed that the size of the cavity has more influence on the periodic trailing-edge flow than its shape does.

Zhdanov and Eckelmann (1994) studied the effect of a cavity at the rear edge of a rectangular plane body with a semicircular leading edge and a blunt rear edge. The frequency of the vortex behind the model was measured by a single-wire probe placed in the external region of the wake and was recorded by a Nicolet 660 frequency spectrum analyzer. The results show that the use of the cavity decreases velocity fluctuations.

Kotsonis et al. (2014) studied influence of circulation around a symmetric airfoil with a rounded trailing edge. Flow control is achieved by the use of dielectric barrier discharge plasma actuators placed at the trailing edge of the airfoil. Time-resolved particle image velocimetry is used to elucidate the topology and dynamical response of the wake flow under the influence of actuation. Flow field measurements indicate the successful manipulation of the Kutta condition enabled by the plasma actuator. The actuator enhanced the mixing of the wake near the trailing edge while reducing the dominant shedding frequency.

Shannon and Morris (2006) studied the velocity field in the near wake region of an asymmetric beveled trailing edge to determine the flow mechanisms responsible for the generation of trailing edge noise. Two component velocity measurements were acquired using particle image velocimetry. The small scale turbulence was found to be dependent on the phase of the vortex shedding process implying a dependence of the broadband sound generated by the trailing edge on the phase of the vortex shedding process.

In this paper, first, the Riso airfoil with a sharp trailing edge is experimentally studied by PIV technique to obtain the flow field around it. Flow structure and vortex dynamics after the separation point are investigated. Then, by cutting off the trailing edge, a thick blunt trailing-edged airfoil is created and experimentally studied by PIV to be compared with the results of the original airfoil. Finally, a base cavity is added to the back of the thick blunt trailing-edged airfoil and its effects on the relevant flow parameters are obtained.

## PIV test setup

An open circuit blower wind tunnel is used with a cross-sectional area of 8 × 8 cm^2^ and length of 1 m. This wind tunnel consists of an air suction blower, a motor from Siemens Company, an inverter from Siemens Company, an air inlet duct, and a honeycomb mesh. The honeycomb mesh is used for establishing a uniform air flow and the inverter for changing the ac motor speed which, in turn, changes the flow velocity. The flow viscosity is 3.6 × 10^−5^ (kg/m s) and free stream velocity changes from 1 to 3 (m/s). Restriction of increasing laser power, camera frame rate, and rate of particles is reason for selection of the test settings, such as flow rate and velocity of free stream.

The PIV method is implemented via a laser-wave company continuous green light laser (wavelength = 532 nm; power = 8 W) and a high-speed PCO camera used for measuring the flow velocity. The laser works at continuous mode. Cool water vapor droplets with the rate of 0.5 kg/h and the size of 1–3 microns are uniformly injected into the air. The density of particles is about 3–6 per 8 × 8 pixel^2^ interrogation area for different measurements. The high-speed PCO camera (Model 1200HS) is used for taking photos of these droplets at a shooting speed of 1800 frames per second. Here, the shooting camera speed is obtained based on flow velocity in the range of 1–3 m/s and maximum search window is equal to 64 × 64 pixel^2^ with 50% overlap. In other words, particle displacement during the time interval between two consecutive images should be less than about 50% of the search window. Here, 50% of the search window is equal to 32 × 32 pixel^2^ in which each pixel is equivalent to 50 microns, and consequently, 50% of the search window becomes equal to 1.6 × 1.6 mm^2^. Therefore, maximum velocity becomes equal to 2.88 m/s which is obtained based on particle displacement between two consecutive images (1.6 mm) divided by its time interval (1800^−1^ s).

The laser power is obtained by trial and error, and should be enough to light the particles during the exposure time of the camera that is less than the inverse of camera speed (fps).

**Fig. 1:**
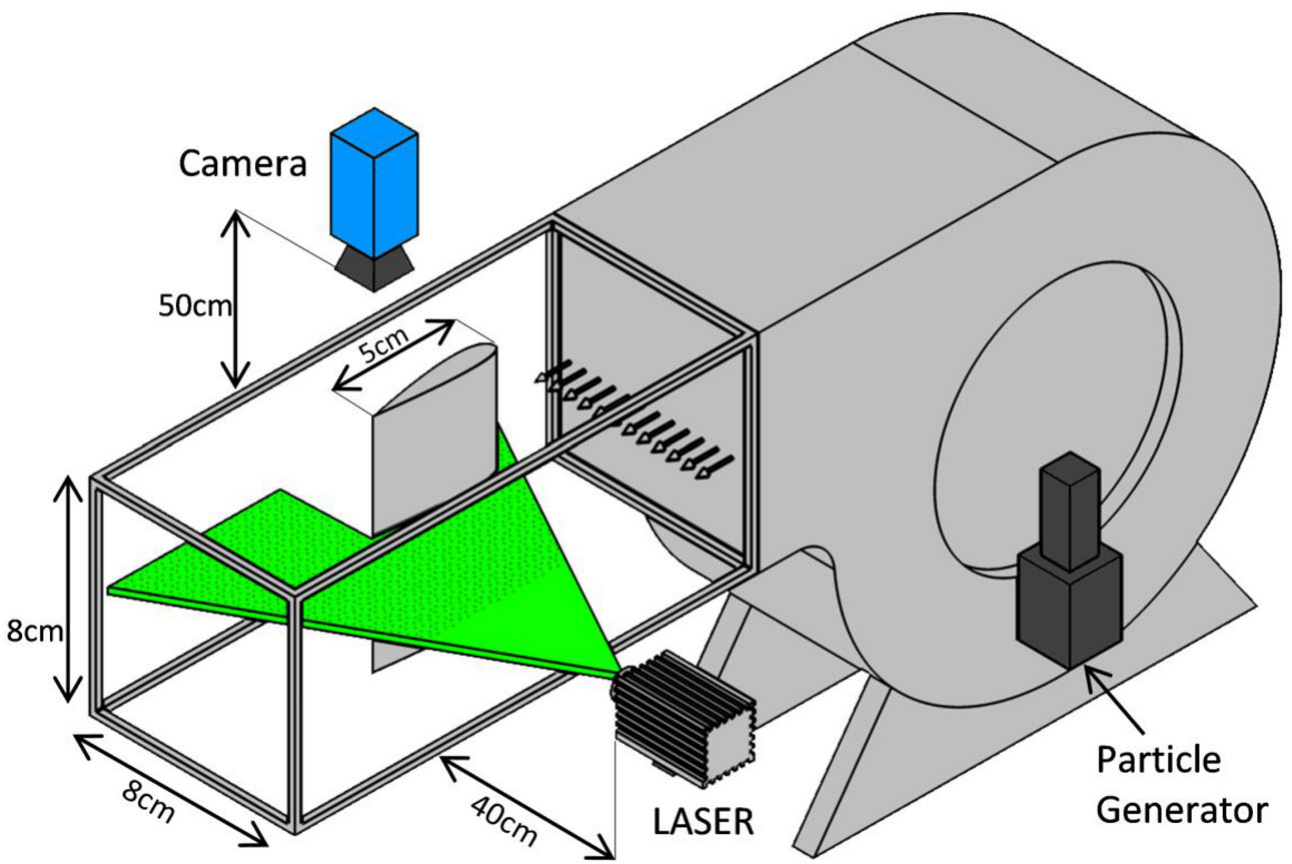
Schematic of the test region in PIV

A VIS–NIR coated cylindrical lens from Edmund Company is placed at the front of the laser to project the laser light onto a plane. The effective focal length of the lens is 20 mm and its dimension is 12.5 × 25 mm^2^. Figure 1 shows the schematic of the test rig of PIV.

**Fig. 2:**
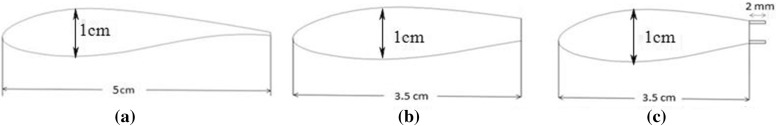
**a** Riso airfoil, **b** thick blunt trailing-edge airfoil, and **c** thick blunt trailing-edge airfoil with a base cavity profile

The Riso airfoil with a chord length of 5 cm and span of 8 cm is placed inside the wind tunnel with the blockage ratio of 12.5%. Maximum thickness of the airfoil is 1 cm. To produce a thick blunt trailing-edge airfoil, the trailing edge of the Riso airfoil is cutoff at a distance of 30% of chord length from its trailing edge. In the last stage, two 2 mm plates are connected to the end of the thick blunt trailing-edge airfoil to form a base cavity behind the airfoil. Figure 2 shows the mentioned profiles used in this study, including: (1) the Riso airfoil, (2) the thick blunt trailing-edge airfoil, and (3) the thick blunt trailing-edge airfoil with a base cavity profile.

In this study, the original airfoil is placed inside the wind tunnel and flow patterns behind the model are measured at different Reynolds numbers and different angles of attack via PIV technique. Then, the same process is repeated for the thick blunt trailing-edge airfoil with and without the base cavity, respectively. Since the flow is laminar, the time scale of the flow pattern is the cord length divided by the flow free stream velocity. In this study, the flow velocity is 1–3 m/s and the cord length is 50 mm, so the time scale is 0.05 s which is one order of magnitude larger than the imaging time sequence.

## Image processing

In this research, velocity vectors are obtained by the PIV lab1.32 software in which FFT algorithm is used for image processing. This algorithm has a high accuracy, especially for flow fields with rotating and stretching movements as well as pure translation. In fact, FFT is a multi-stage method in which the interrogation window is reproduced at each stage to be capable of capturing rotating and stretching movements (Gilbert and Johnson 2003). Here, 32 × 32 pixel^2^ interrogation window for the first stage, 16 × 16 for the second one, and 8 × 8 for the last one are used. In each stage, 50% overlap is applied; thus, the computation error is 1 pixel. By considering the blockage length of the model for image calibration, each pixel is equivalent to 50 microns.

## Uncertainty and validation

**Fig. 3:**
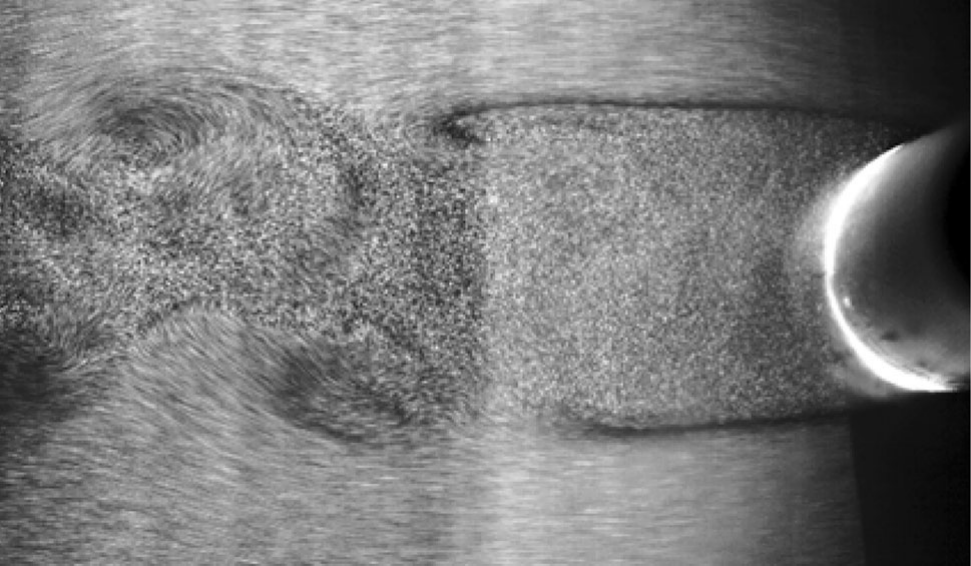
Flow pattern behind the cylinder

Uncertainty in PIV method can be divided into two main parts: first, the general errors due to tracer particles lag and their not completely following from the flow pattern, and the second one, the error of image processing. The uncertainty of PIV image processing methods was investigated by McKenna and McGillis (2002). They noted that the uncertainty increases by increasing the size of interrogation window. On the other hand, increasing the size of interrogation window causes the spatial resolution of velocity field to decrease. Their calculations showed that for 32 × 32 pixel^2^ interrogation window and FFT algorithm, the uncertainty of velocity measurement is 1.93% (McKenna and McGillis 2002).To verify the PIV test results, Strouhal number is calculated from PIV test results at three Reynolds numbers of 1500, 2000, and 2500, and then compared with the results of Rahman et al. (2007). Figure 3 shows the raw image captured from this experiment. Table 1 shows that the maximum error is 2%. The error is calculated from the following equation:

1 $${\text{Error}}\;\% = \frac{{{\text{Strouhal}}\; ( {\text{current work)}} - {\text{Strouhal}}\;({\text{Rahman et\,al}}.\;2007)}}{{{\text{Strouhal }}\;({\text{current work}})}}.$$

**Table 1:** Comparison of Strouhal number between this work and (Rahman et al. 2007)

Error %	Strouhal (Rahman et al. 2007)	Strouhal (current work)	Reynolds number
0.9	0.218	0.22	1500
1	0.2178	0.22	2000
2	0.2176	0.222	2500

In addition, the experiments were repeated for three times and results show that the difference between them is less than 5%.

## PIV results for the original airfoil

First, the flow around the original airfoil was studied. Next, the flow around the airfoil with a thick blunt trailing-edge was compared with that around the original airfoil. Finally, the effect of adding the base cavity to the thick blunt trailing-edge airfoil was studied.

**Fig. 4:**
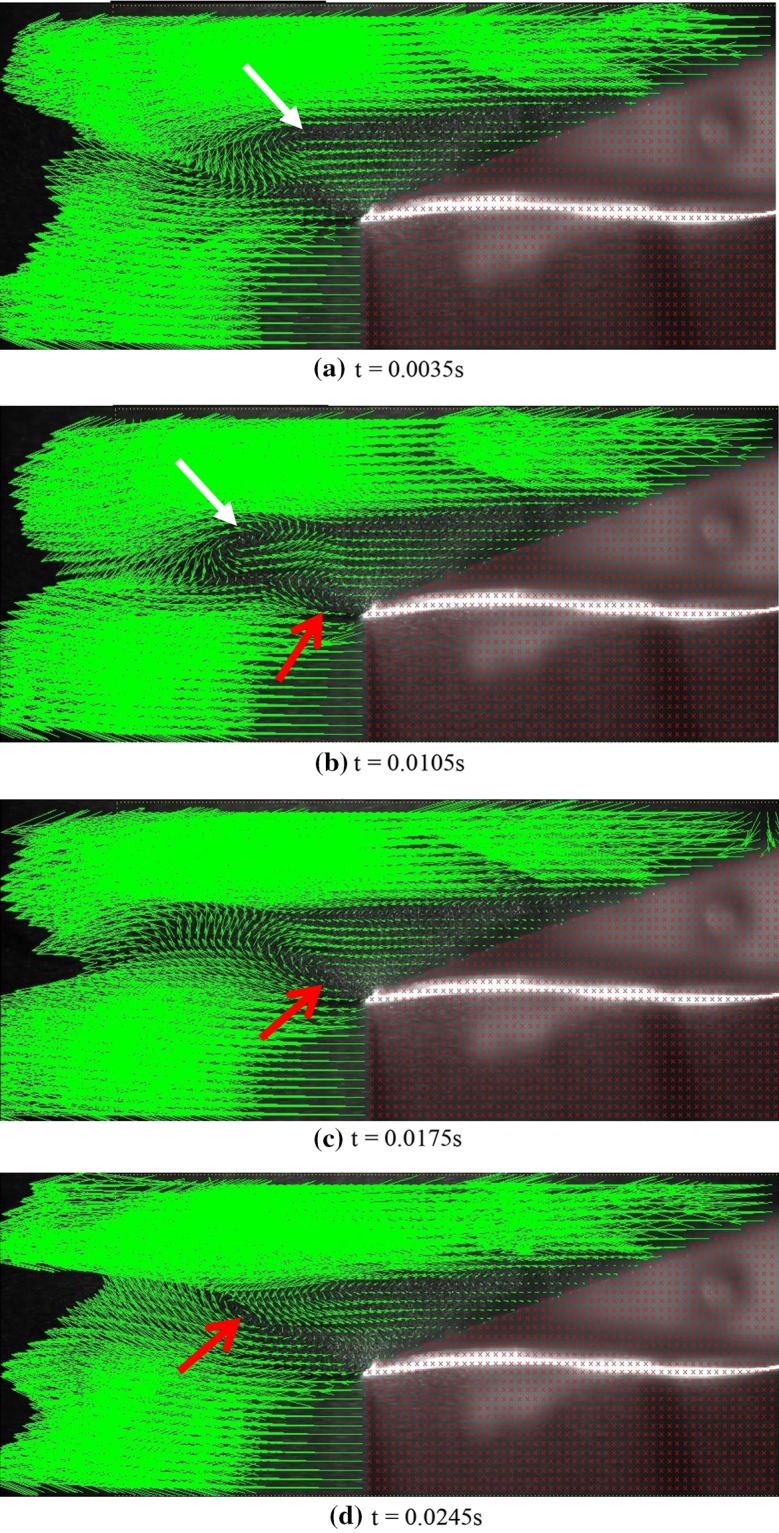
Instantaneous velocity vectors over the original airfoil at various instances at *Re* = 2150, *U* = 1 m/s, and attack angle = 5°

Figure 4 shows the instantaneous velocity vectors obtained during a specific time interval for the original airfoil. The temporal distance between two consecutive images is 0.007 s. The selected time sequence should be enough to encompass the period of flow and to show the entire of flow pattern. Since the flow is laminar, the time scale of the flow pattern is the flow length scale which is the cord length divided by the flow free stream velocity. In this study, the flow velocity is 1 m/s and the cord length is 50 mm, so the time scale is 0.05 s which is one order of magnitude larger than the imaging time sequence. Angle of attack and Reynolds number are 5° and 2150, respectively. A dark region is created under the airfoil resulting from refraction as laser light hits the airfoil, rendering image processing and velocity measurements impossible within this region. The red regions actually show the airfoil and its underneath area excluded from the images. Analysis of the dark region under the airfoil can be made possible by projecting another laser onto this region. As observed in this figure, the flow experienced separation upon passing over the airfoil, creating a recirculation region with vortex shedding on the airfoil. First, a vortex is formed within the separation region near the upper shear layer. This vortex rotates in the counter clockwise direction and marked with a white arrow in the images. Initially, the vortex separated from the shear layer and moves with the flow, and then, it disappears within a short time after passing the airfoil. However, while the first vortex is disappearing, a second vortex rotating clockwise (the red arrow) starts to form at the trailing edge near the lower shear layer. This vortex also separated from the lower shear layer and moves with the flow before disappearing completely. The direction of motion of this vortex is perpendicular to the airfoil surface. The above phenomenon is continuously repeated at a specific frequency and is what we call “vortex shedding”.

## PIV results for the thick blunt trailing-edge airfoil

**Fig. 5:**
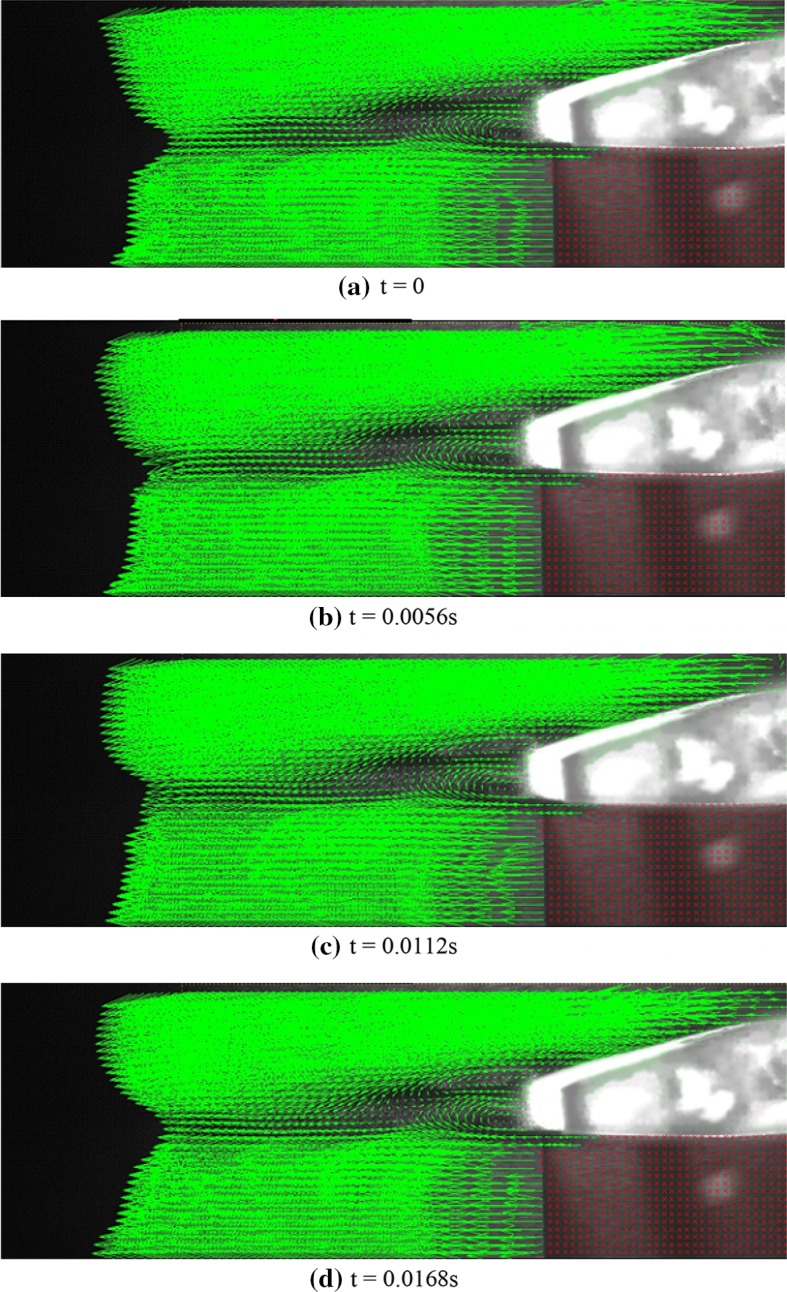
Instantaneous velocity vectors over the thick blunt trailing-edge airfoil at various instances and *Re* = 2150, *U* = 1 m/s, and attack angle = 5°

Keeping Reynolds number and attack angle constant (*Re* = 2150 and attack angle = 5°), the PIV tests are performed again for the thick blunt trailing-edge airfoil. Figure 5 shows the instantaneous velocity vectors obtained for this case. The time frame between two consecutive images is 0.0056 s. As seen, no separation occurs on the airfoil surface, and only two vortices are formed behind thick blunt trailing edge. In fact, using this thick blunt trailing-edge airfoil causes the location of the separation region to move from the airfoil surface to the back of the airfoil. No further vortex shedding is observed at this point and, as said before, only two vortices are formed at the upper and lower parts of the airfoil with counter clockwise and clockwise rotations, respectively.

**Fig. 6:**
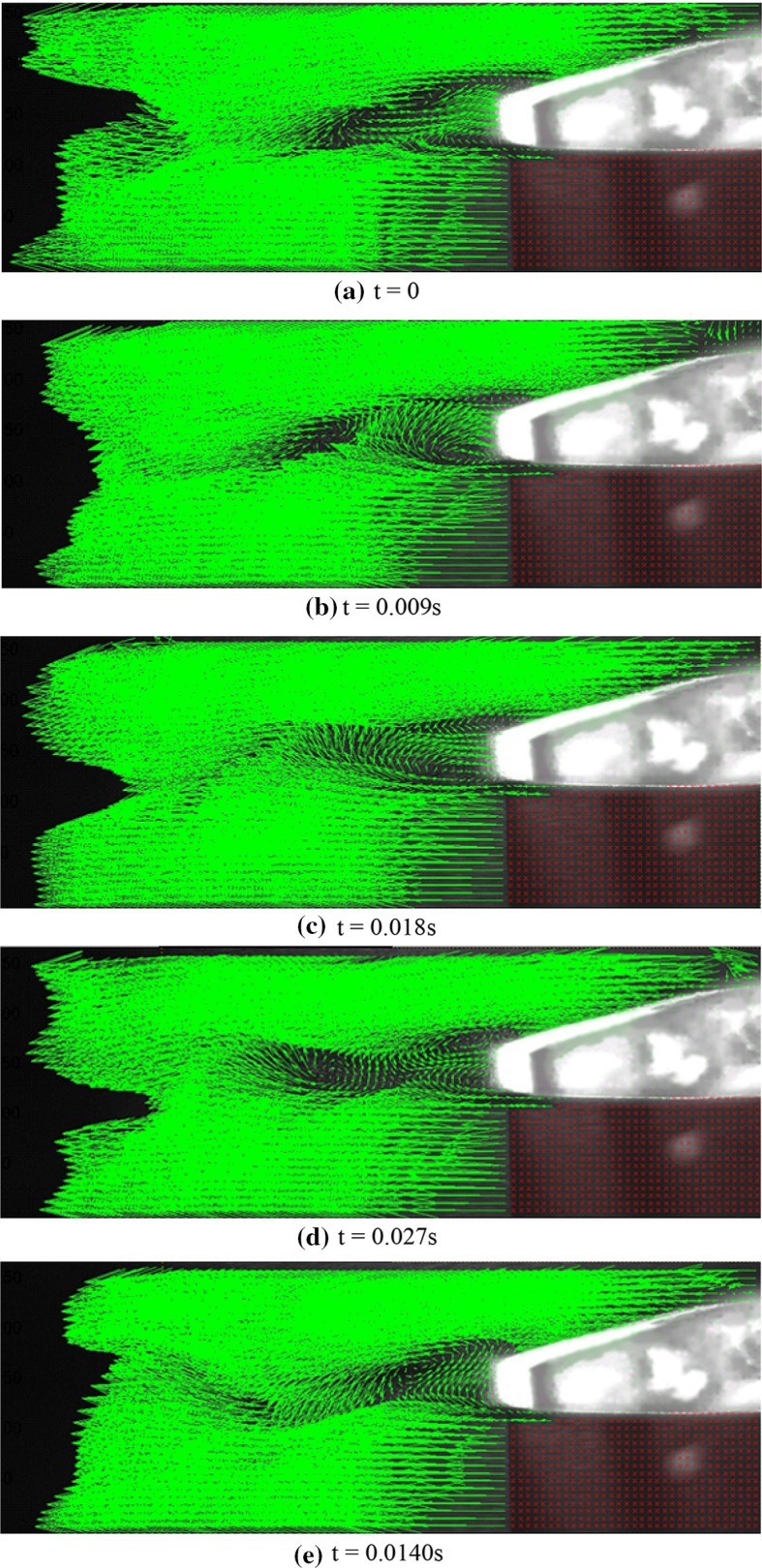
Instantaneous velocity vectors for flow over the thick blunt trailing-edge airfoil at various instances for *Re* = 3220, *U* = 1.5 m/s, and attack angle = 5°

Flow study at higher Reynolds number (*Re* = 3220) on the thick blunt trailing-edge airfoil shows that as the Reynolds number increases, the vortices (unlike the *Re* = 2150 case) no longer remain in a constant location, but move at a specific frequency along the flow as explained here. First, a counter clockwise vortex is separated from the upper shear layer and starts moving with the flow before disappearing towards the end of the separation zone. A second vortex (rotating clockwise) is immediately separated from the lower shear layer, again moving with the flow until it vanishes. This is periodically repeated at a specific frequency. Figure 6 shows the instantaneous velocity vectors for a vortex shedding time period at *Re* = 3220 and attack angle of 5°. The time between two consecutive images is 0.009 s. As observed, in the first two images, the upper vortex, rotating counter clockwise, is moving in the same direction as the free stream. In the third image, this vortex is disappearing and a second vortex is being formed at the lower part, rotating in a clockwise direction. In the fourth image, the lower vortex is fully formed and is moving in the direction of the free flow. In the fifth image, this vortex is also disappearing and a third upper vortex is being formed. Finally, the last image shows that the lower vortex fully vanishes and the upper vortex is fully formed, and, thus, the cycle is completed.

## Velocity distributions behind the trailing edge

**Fig. 7:**
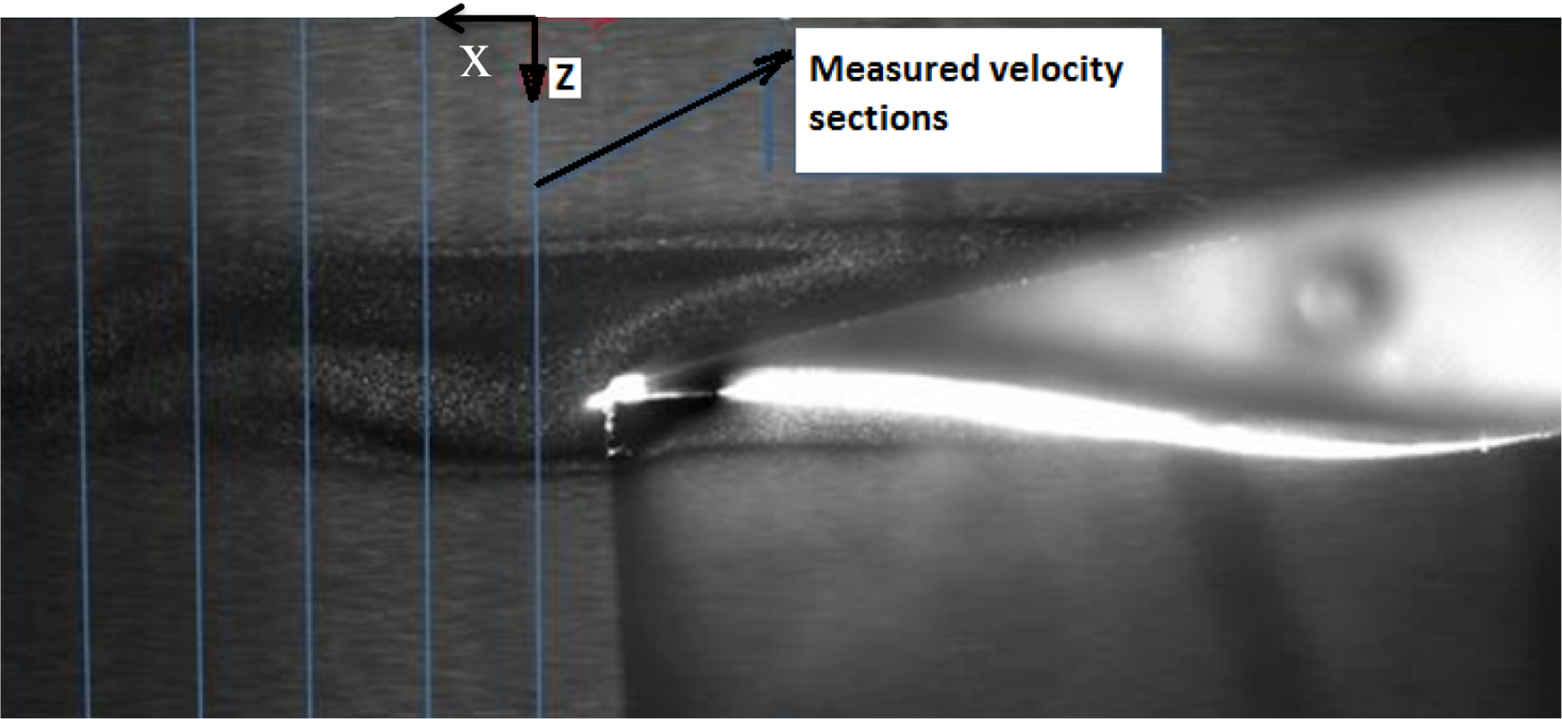
Sections behind the airfoil for measurement of flow velocity distribution

In Fig. 7, five sections at the back of the airfoil trailing edge have been specified along which velocity distributions are plotted. These five sections are located at 10, 20, 30, 40, and 50% of the chord length (5 cm) from the trailing edge.

**Fig. 8:**
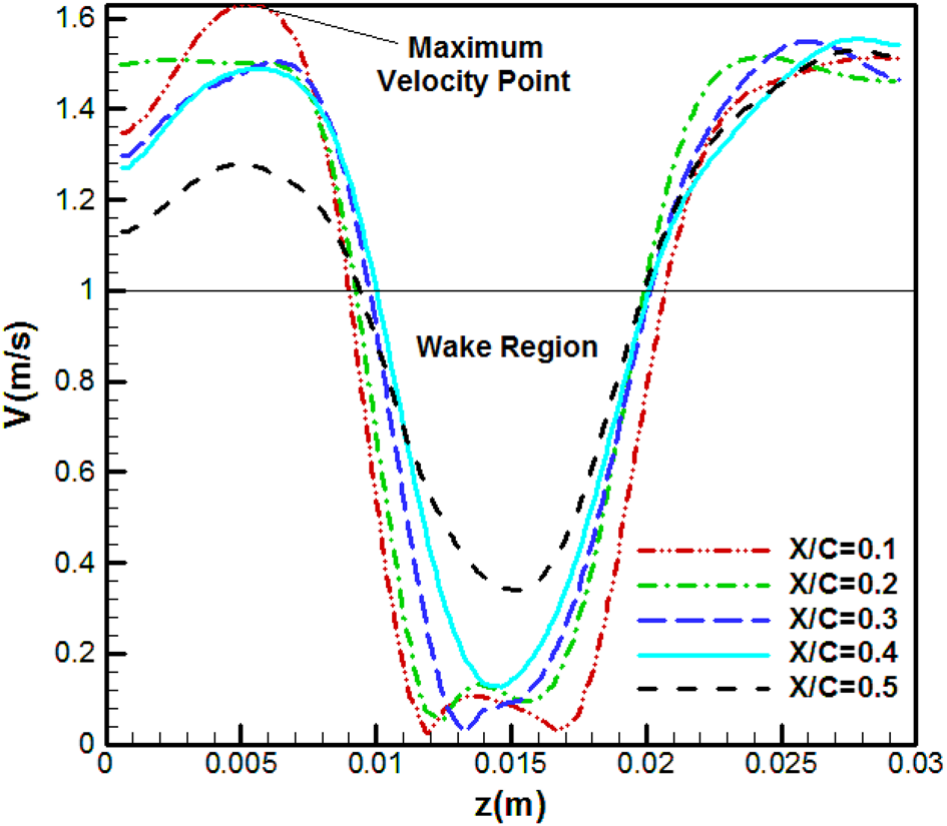
Velocity distribution at various sections of the original airfoil at a 5° angle of attack and *U* = 1 m/s

Figure 8 shows the mean velocity distributions along the five specified sections at the attack angle of 5° and the Reynolds number of 3070. The mean velocity distributions are averaged by taking the mean values for several vortex shedding periods. The coordinate origin coincides with the upper point of the diagram. As can be observed, at the initial sections (10 and 20% of the chord), there are two minimum points in the diagram. These points appear due to the formation of two vortices within the separation region. These vortices are the same as those formed during the vortex shedding (in the previous section) which occurred near the upper and lower shear layers. As we move further from the trailing edge, the effects of these two vortices diminish and the two minimum points are consequently merged. As the distance of the section from the end point is further increased, this recirculation region gradually vanishes. The close region between the black line (1 m/s) and the lower part of the velocity curves shows the recirculation or wake region.

In Fig. 8, there is a maximum point above the airfoil. As shown in this figure, the flow velocity increases up to a certain point and then decreases. As can be seen, such a point does not exist under the airfoil. As the flow is separated on the airfoil, the free flow above the shear level is accelerated and flow velocity at the upper shear layer increases. Therefore, the maximum point is due to this acceleration in the proximity of the upper shear layer. This phenomenon can be explained as follows: the flow first accelerates while passing over the upper airfoil surface before encountering a positive pressure gradient. However, upon flow separation, full pressure recovery does not occur. Therefore, we expect the flow velocity in the upper part of the separated region to exceed that of the free air flow velocity. The same situation does not exist under the airfoil, since velocity does not increase on the lower surface of the airfoil.

**Fig. 9:**
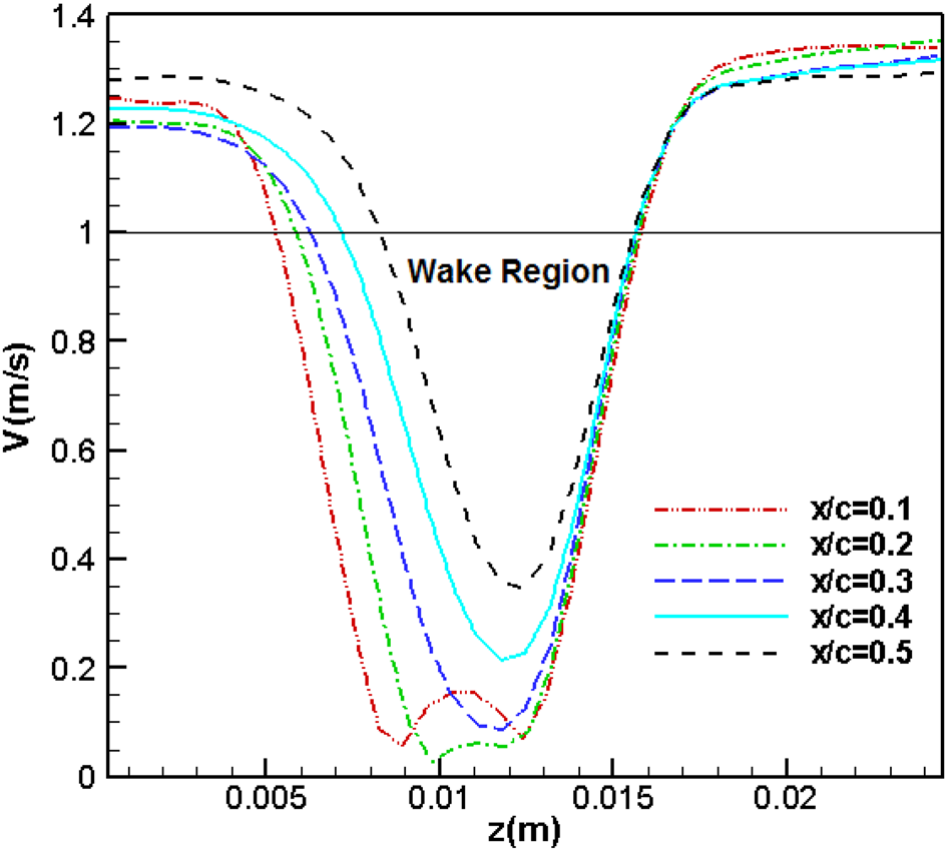
Velocity distribution at various sections of the thick blunt trailing-edge airfoil (angle of attack = 5° and *U* = 1 m/s)

Figure 9 shows the flow velocity profiles at various sections of the thick blunt trailing edge airfoil for the Reynolds number of 2150 and attack angle of 5°. The chord length for this airfoil is 3.5 cm. As seen, the maximum velocity point that exists in Fig. 9 no longer appears and the flow velocity remains constant upon reaching its maximum value. As was mentioned above, the flow over the thick blunt trailing edge airfoil, positioned at a 5° angle of attack, does not undergo separation and this is why a maximum velocity point is not observed in this case. As the angle of attack increases, we observe a recurrence of separation and the reappearing of this maximum velocity point. Comparison between Figs. 8 and 9 shows that by getting further from the trailing edge, the wake region behind the blunt trailing edge airfoil decreases more than that behind the original airfoil. It indicates that the wake region behind the thick-edged airfoil is disappearing faster than that behind the original one.

## PIV results for the thick blunt trailing-edge airfoil with base cavity

As mentioned above, deploying a base cavity is one way of reducing drag in an airfoil with a thick blunt trailing edge. In this study, we add a base cavity to the airfoil upon thickening its edge, and study the effects thereof on the flow over the airfoil.

**Fig. 10:**
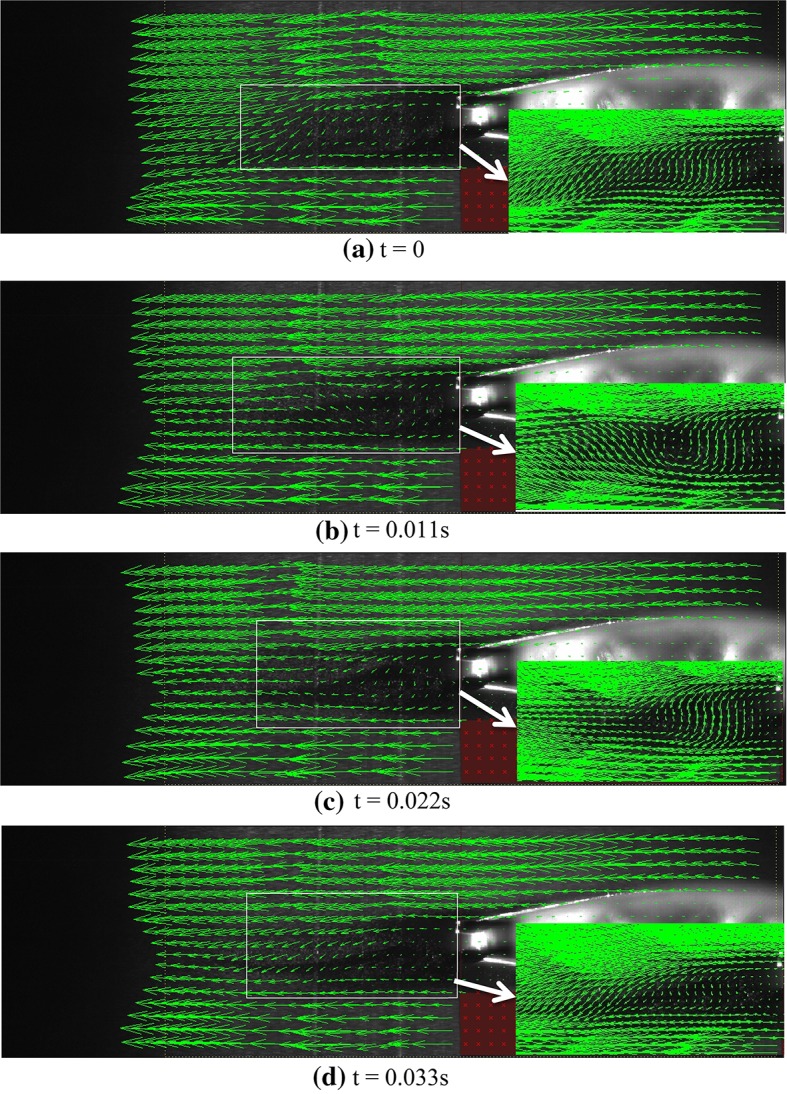
Instantaneous velocity vectors for flow over the thick blunt trailing-edge airfoil with base cavity at various instances for angle of attack = 5° for *Re* = 2150 and *U* = 1 m/s

Figure 10 shows the instantaneous velocity vectors for the thick blunt trailing-edge airfoil connected to a base cavity (angle of attack = 5° and *Re* = 2150). Similar to the case without base cavity, no separation occurs here either. It is obviously observed here that the separation region behind the airfoil has changed its shape into a pointed triangle, and that the separation region is much smaller than that observed when no cavity was used. In the absence of a cavity, two distinct vortexes appeared behind the thick trailing edge, whereas here, no such vortices are visible.

Due to the use of dark-colored blades, the flow inside the cavity could not be observed. The reason why dark blades are used is that they prevent light reflection which would distort the images. Thus, in the absence of direct observation from inside the cavity, we can conclude that the two vortices move into the cavity when a base cavity is connected to the airfoil.

**Fig. 11:**
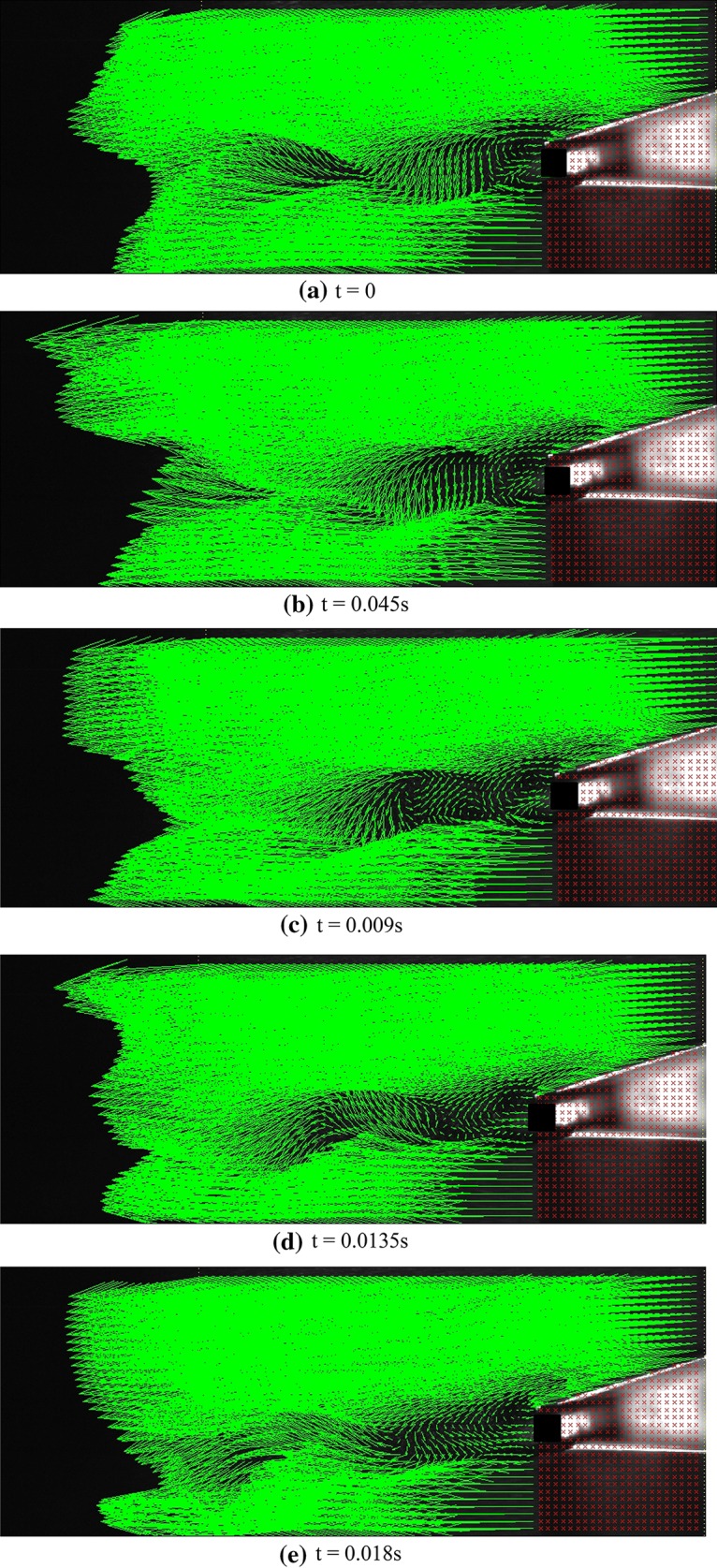
Instantaneous velocity vectors for flow over the thick blunt trailing-edge airfoil with base cavity at various instances for attack angle of 5° and *Re* = 3070, *U* = 1.5 m/s

As the Reynolds number increases, the momentum also increases, leading to the instability of these vortices. Thus, the vortices exit the cavity and become observable. Although these vortices are much smaller than those observed when no cavity is used, they are, nevertheless, observable and move periodically along the flow line. Figure 11 shows the instantaneous velocities for one period of oscillation. In the first image, a vortex is being formed at the upper edge, and in the second image, this vortex is being separated from the upper edge of the cavity. In the third image, the vortex has fully separated from the cavity edge and is moving along the flow line. At the same time, a second vortex (rotating in a clockwise sense) is formed and separated from the lower edge. The upper vortex in the fourth image has fully disappeared, whereas the lower vortex is still moving with the flow. Finally, in the last image, the second vortex also vanishes.

## Size of separation region

**Fig. 12:**
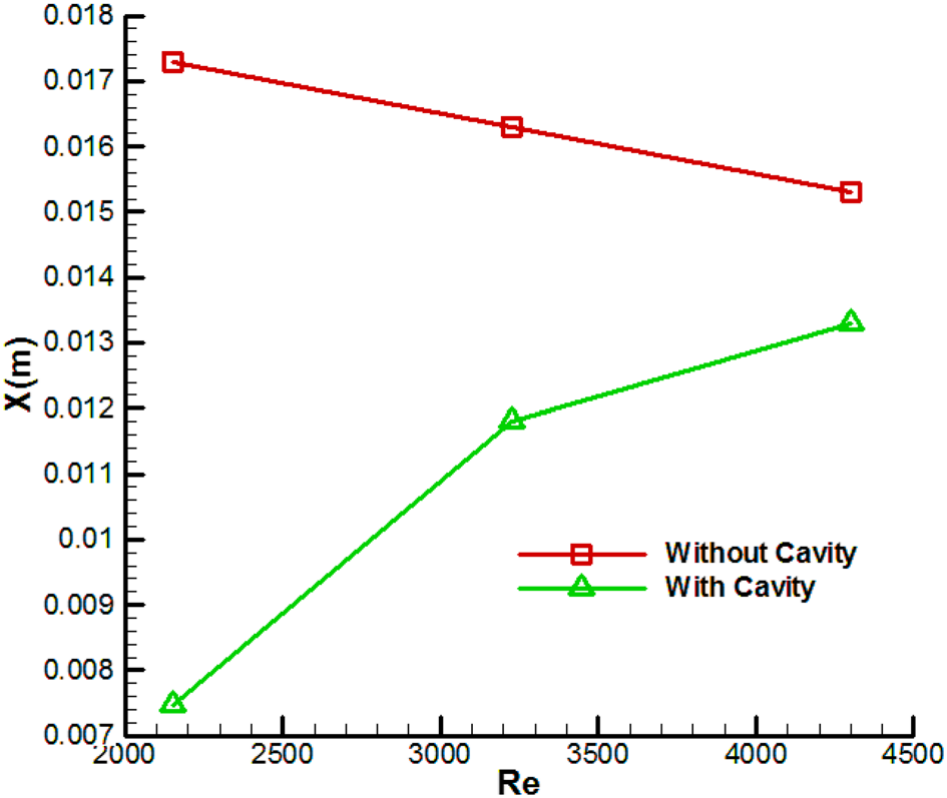
Size of flow separation region (*x)* for the thick blunt trailing-edge airfoils with and without the base cavity at various Reynolds numbers

Figure 12 shows the size of the separation region for the thick blunt trailing-edge airfoil with and without base cavity at attack angle of 5° and various Reynolds numbers. As observed, the size of the separation region was smaller in the case with the base cavity than that without it. This can be indicative of the positive effect of using a base cavity. However, another noteworthy point is that in the presence of a base cavity, the separation region length increases with increasing Reynolds number; whereas the separation region length would decrease in the absence of a base cavity. The reason is that: at low *Re*, the vortices are sucked into the base cavity and are thus stabilized. As *Re* increases, the flow momentum overcomes the effects of the base cavity and, as a result, the vortices, having thus been destabilized, move out of the cavity. Vortex instability actually increases with increasing *Re*, causing the effects of the base cavity to diminish.

**Fig. 13:**
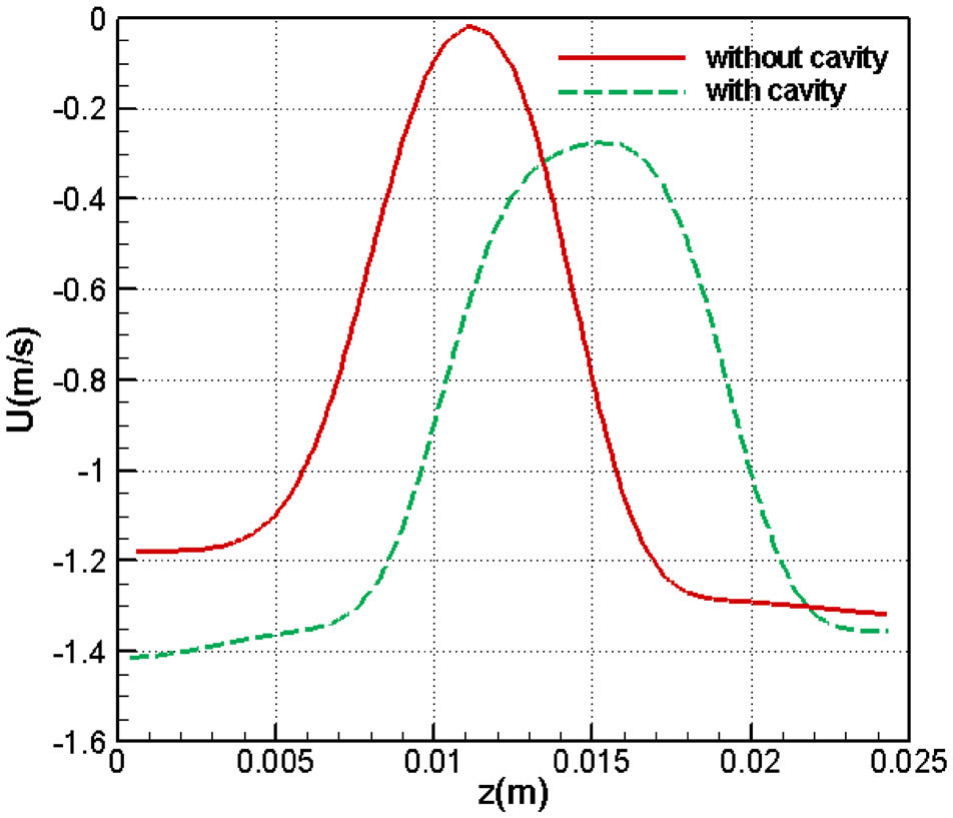
Horizontal velocity component for a section located at 0.40% of the chord length form the trailing edge (angle of attack = 5° and *Re* = 2151)

In Fig. 13, the horizontal velocity component along the vertical section is given for the thick blunt trailing-edge airfoil with and without the base cavity. The distance from the airfoil trailing edge is equal to 40% of the chord length, and the Reynolds number and attack angle are equal to 2150° and 5°, respectively.

Using the base cavity has both positive and negative effects. The positive effect is that in the presence of base cavity, the minimum velocity inside the separation region is increased and this is indicative of higher flow momentum and lower drag. However, the negative effect is that, as seen from the diagram in Fig. 13, the width of the parabola is increased after adding the base cavity. This means that the separation region width has also increased and this is unfavorable, since it leads to an increase in drag. To determine the percentage of drag reduction upon adding the cavity, we notice that increase in momentum behind the airfoil is equal to decrease in drag. The flow momentum behind the airfoil is obtained from ($\rho l\int {u^{2} {\text{d}}z}$), where (*l*) is the width of the duct. As (*ρl*) is constant, we conclude that if ($\int {u^{2} {\text{d}}z}$) is increased by a certain amount, drag would decrease by the same amount. To demonstrate the effect of both these factors on drag force, we calculated ($\int {u^{2} {\text{d}}z}$) for the two different cases, namely, in the presence of the base cavity and in the absence thereof. For the former case, we obtained ($\int {u^{2} {\text{d}}z}$) equal to 0.02673 and for the latter case equal to 0.023509. These values show that when the base cavity is used, momentum at the outlet increases by 13.7%, leading to a drag reduction.

## Effect of using cavity on Strouhal number

**Fig. 14:**
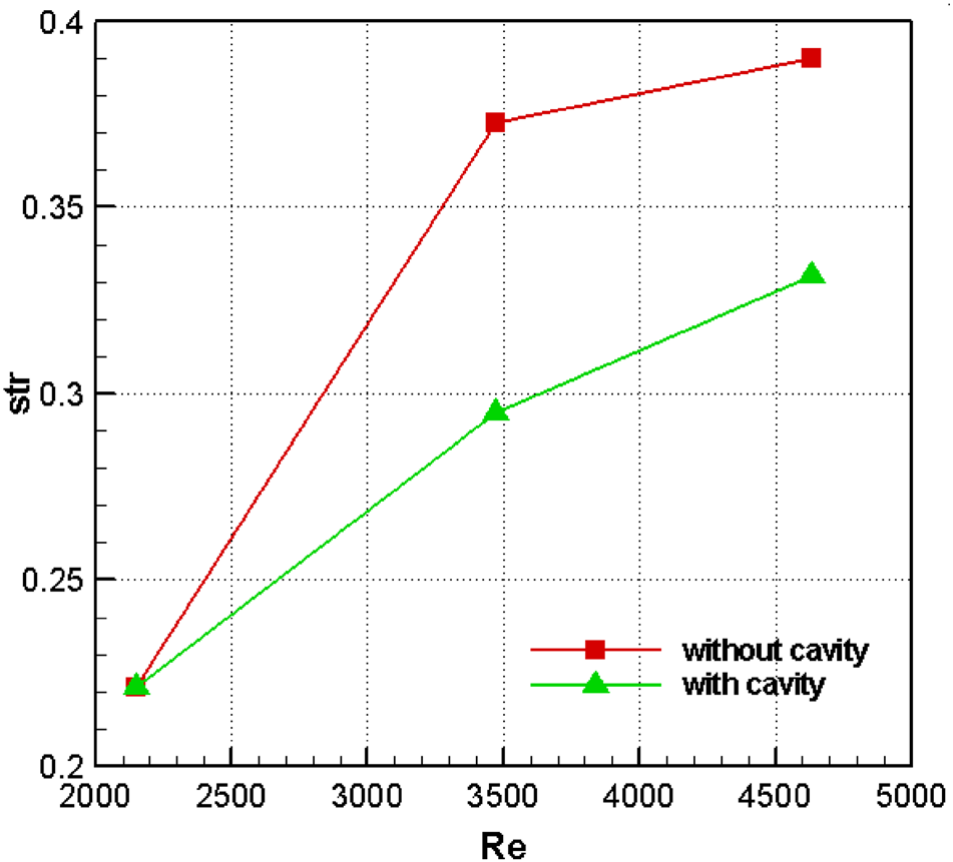
Strouhal number vs. Reynolds number for the thick blunt trailing-edge airfoils with and without cavity at a 10° angle of attack

Vortex shedding is a problem encountered in the flow around thick blunt trailing-edge airfoils, causing noise as well as oscillatory forces which damage airfoil. This problem is reduced at lower frequencies. The characteristic length for calculating the Strouhal number is assumed to be equal to the amount of flow blockage. Figure 14 shows the diagram of Strouhal number versus Reynolds number for the thick blunt trailing-edge airfoils with and without cavity at the attack angle of 10°. As can be seen, the Strouhal number is lower for the airfoil with cavity. In fact, this diagram clearly shows the positive effect of the cavity in reducing the frequency of the vortex shedding phenomenon. This effect cannot be properly demonstrated at lower *Re* values (e.g. *Re* = 2000). However, it becomes more prominent as *Re* increases.

## Conclusion

In this study, the PIV technique was continuously used to study the flow velocity field around three types of airfoils, including an original thick airfoil with sharp trailing edge, a thick blunt trailing-edge airfoil created from cutting off the original airfoil, and the thick blunt trailing-edge airfoil with a base cavity. Investigation of unsteady flow field behind Riso airfoil with thick blunt trailing edge and base cavity has not been accomplished yet. Here, using PIV method allows viewing fluid flow at short intervals behind the models.

For the original Riso airfoil when flow was separated over its suction surface, due to the velocity difference between the separation region and the free flow region, two (upper and lower) high velocity gradient regions developed next to the separation region. A clockwise and a counter clockwise vortex developed in the separation region that are alternately generated and vanished.

In the second case, cutting off the end of the original airfoil decreased the wake region behind the airfoil and delayed the separation. In fact, separation did not occur on this case, and instead, two vortices developed in the upper and lower regions behind the thick blunt trailing edge, rotating in the CW and CCW directions, respectively. However, increasing the Reynolds number caused the length of the wake to increase.

Finally, a base cavity was added to the thick blunt trailing-edge airfoil to further decrease in drag. Upon using a base cavity, the two vortices were sucked into the cavity. This somewhat decreased the size of the separation region. Increasing the Reynolds number caused the vortices to be exited from the cavity, thus increasing the length of the separation region as well as the recirculation zone. Moreover, using the base cavity decreased vortex shedding frequency, leading to a lower Strouhal numbers at the attack angle of 10°. Comparison between the momentum behind the thick blunt trailing-edge airfoil with and without the base cavity revealed that the base cavity increased the momentum, leading to a drag reduction.
